# Supplementary material for: DiTEC-WDN: A Large-Scale Dataset of Hydraulic Scenarios across Multiple Water Distribution Networks
Source: Sci Data. 2025 Nov 3;12:1733. doi: 10.1038/s41597-025-06026-0 (PMC12583744; doi:10.1038/s41597-025-06026-0)
Supplement: Supplementary file 1 — Supplementary Table S1. List of collected WDNs. [file 41597_2025_6026_MOESM1_ESM.pdf]

1 Supplementary Information

WDN	Description	Junctions	Pipes	Reservoirs	Tanks	Pumps	Patterns
ky1	Synthetic Water Distribution Networks (WDNs) based on a statewide database of water systems originally developed by the Kentucky Infrastructure Authority, in United States.	856	985	1	2	1	2
ky2		811	1125	1	3	1	3
ky3		269	371	3	3	5	3
ky4		959	1158	1	4	2	3
ky5		420	505	4	3	9	3
ky6		543	647	2	3	2	4
ky7		481	604	1	3	1	4
ky8		1325	1618	2	5	4	4
ky10		920	1061	2	13	13	4
ky13		778	944	2	5	4	3
ky14		377	553	4	3	5	3
ky16		791	915	3	4	7	3
ky18		772	917	4	0	3	9
ky24_v		288	292	2	0	0	3
19 Pipe System	An artificial WDN with two sources. It was used for analyzing flow distribution in hydraulic networks.	12	21	2	0	0	3
Anytown	It is a hypothetical WDN used as part of a Battle of the Networks competition aimed at improving analysis methods.	19	41	3	0	1	1
new_york	It represents the water supply transmission tunnels for the City of New York in 1969. It was originally used to optimize duplications to the existing system to meet projected demand increases.	19	42	1	0	0	4
Jilin	It is a synthetic network used as part of a study of optimization of WDNs via online retrained metamodels.	27	34	1	0	0	1
hanoi	This WDN is based on the planned trunk network of Hanoi, Vietnam. It was originally used to test pipe size optimization software.	31	34	1	0	0	0
fossolo (foss_poly_1)	It is based on the WDN of the Fossolo neighborhood in Bologna, Italy. It was used for WDN design optimization.	36	58	1	0	0	0
FOWM	It is a skeletonized version of the WDN of northern Arlington County in the United States.	44	49	1	0	0	0
EPANET Net 3	It is based on the North Marin WDN in Novato, California. It was used as part of a water quality study.	92	119	2	3	2	5

Continues on Next Page…

WDN	Description	Junctions	Pipes	Reservoirs	Tanks	Pumps	Patterns
FFCL-1	It is based on the Fairfield WDN, a relatively small system with a single source. It was originally used to study numerical modeling methods for water quality.	111	126	0	1	0	3
Zhi Jiang (ZJ)	It is a simplified version of the Zhi Jiang WDN in the eastern province of China. It was originally used as part of a design and optimization study.	113	164	1	0	0	0
WA1	It is based on the Bellingham WDN in Washington, US. It was originally used for water quality modeling.	121	169	0	2	0	6
OBCL-1	It is based on the Cheshire WDN located near Harrisburg, Pennsylvania. It was used originally to study the kinetics of chlorine decay.	262	289	1	0	1	5
modena	It is a simplified version of the WDN of the town of Modena, Italy. It was originally used for WDN design studies.	268	317	4	0	0	0
NPCL-1	It is based on the North Penn Water Authority WDN. It was originally used for water quality studies.	337	399	0	2	0	17
Marchi Rural (RuralNetwork)	This WDN was adapted from an irrigation system in Australia. It was originally used as part of a design and optimization study.	379	476	2	0	0	0
CTOWN	It is based on a real small WDN, the data was in part obtained from a geographic information system of the Municipality of C-Town, and a part from SCADA systems.	388	444	1	7	11	5
d-town	It is a hypothetical WDN created as part of a Battle of the Networks focused on long term improvement plans that account for greenhouse gas emissions.	399	459	1	7	11	5
balerma	This WDN is an adaptation of an existing irrigation network in the Sol-Poniente irrigation district, located in Balerma in the province of Almería in Spain.	443	454	4	0	0	0
L-TOWN	It is a synthetic WDN, based on a real WDN of a city in Cyprus. It was created for the Battle of Leakage Detection and Isolation Methods (BattLeDIM).	782	909	2	1	1	107

Continues on Next Page…

Supplementary Table S1 – Continued

WDN	Description	Junctions	Pipes	Reservoirs	Tanks	Pumps	Patterns
KL	It is a synthetic WDN, originally used in a study on the heuristic hierarchical approach to optimization of WDN design.	935	1274	1	0	0	0
Exnet (EXN)	It is a synthetic WDN proposed by the Centre for Water Systems of Exeter University. It was created as a benchmark in multi-objective optimization problems.	1891	2467	2	0	0	0
Large	It is a hypothetical WDN based on a real data. It has one source node, which supplies the entire WDN. It was used originally for design optimization.	3557	4021	1	0	0	0

Supplementary Table S1. List of collected WDNs.
